# Groin Surveillance by Serial Ultrasonography Rather Than Sentinel Node Biopsy or Inguinofemoral Lymphadenectomy for Patients with Vulvar Cancer: A Pilot Study

**DOI:** 10.3390/cancers15030831

**Published:** 2023-01-29

**Authors:** Neville F. Hacker, Ellen L. Barlow, Glenn McNally, Stephen Morrell, Val Gebski, Andreas Obermair

**Affiliations:** 1 School of Women’s & Children’s Health, University of New South Wales, Sydney 2052, Australia; 2 Gynaecological Cancer Centre, Royal Hospital for Women, Sydney 2031, Australia; 3 Medical Imaging Department, Royal Hospital for Women, Sydney 2031, Australia; 4 School of Population Health, University of New South Wales, Sydney 2052, Australia; 5 Cancer Institute New South Wales, New South Wales Ministry of Health, Sydney 2065, Australia; 6 NHMRC Clinical Trials Centre, University of Sydney, Sydney 1450, Australia; 7 Queensland Centre for Gynaecological Cancer, Royal Brisbane & Women’s Hospital, Brisbane 4102, Australia; 8 Centre for Clinical Research, University of Queensland, Brisbane 4072, Australia

**Keywords:** vulvar cancer, groin dissection, ultrasonic surveillance

## Abstract

**Simple Summary:**

Vulvar cancer is a rare disease, but its treatment often causes significant issues with body image. One such issue is chronic lower limb lymphedema, which commonly occurs following the resection of groin lymph nodes. The status of these nodes is the most important prognostic factor for patients with vulvar cancer, and all patients but those with small, superficially invasive cancers currently require some form of groin dissection. If patients recur in an undissected groin, there is a 90% mortality rate. We have hypothesized that it may be possible to avoid groin dissection in selected patients by performing serial ultrasonography of the groin for at least 12 months. In this pilot study on 32 patients, we were able to identify three patients (9.4%) with positive nodes upon ultrasound. After groin dissection and radiation therapy, one patient (3.1%) died while 90.7% of groins were preserved intact.

**Abstract:**

A pilot study was conducted to determine whether 3-monthly groin ultrasonography could eliminate groin dissection after a negative bilateral groin ultrasound in three groups of patients: (i) Those with a unifocal stage 1B squamous cell carcinoma of up to 20 mm in diameter. (ii) Those with an ipsilateral squamous cell carcinoma of any size which extended to within 1 cm either side of the midline. These patients underwent ipsilateral inguinofemoral lymphadenectomy and ultrasonic surveillance of the contralateral groin. (iii) Patients with multifocal invasive lesions with the largest individual focus 20 mm or less in diameter. Three additional patients were added because they either refused groin dissection or were considered unfit for surgery. All ultrasonically positive nodes were confirmed histologically. Thirty-two patients were entered, and no patients were lost to follow-up. Forty-three groins were followed. With a median follow-up of 37 months, three positive nodes (9.4%) were detected. One patient died of her recurrence (3.1%), and 39 groins (90.7%) were preserved. The overall sensitivity of ultrasonic surveillance was 100% (95% CI: 44–100%), with a specificity of 97% (95% CI: 83–99%) and a negative predictive value of 100% (95% CI: 88–100%). This pilot justifies a larger study on serial ultrasonography in lieu of groin dissection in selected patients with vulvar cancer.

## 1. Introduction

Vulvar cancer is a rare disease. In the United States in 2018, 5496 women (2.6 per 100,000) were diagnosed with the disease, and 1316 women (0.6 per 100,000) died from it [1].

The status of the groin lymph nodes is the most important prognostic factor, and all patients except those with stage IA disease presently require some form of groin node dissection [2]. Patients with more than one positive node, or those with extracapsular spread, will benefit from adjuvant postoperative groin and pelvic radiation therapy [3,4].

Groin node dissection is associated with both short- and long-term morbidity. The most frequent perioperative complication is lymphocyst formation, which occurs in about 40% of cases after complete inguinofemoral lymphadenectomy. Other common acute complications include groin wound breakdown and groin cellulitis [5,6,7]. The major long-term morbidity is lower limb lymphedema. The reported incidence of clinically significant lymphedema ranges from 10.9% [8] to 67% [9], and both the incidence as well as severity are related to the number of lymph nodes removed [5].

The imaging of groin nodes has not been extensively studied [10]. Computerized tomographic (CT) and magnetic resonance imaging (MRI) scans both rely on the size of the lymph node, such that occult metastatic disease in normal-sized nodes is unlikely to be detected [11,12].

The 18F-FDG PET/CT scan is expensive and is of little value for the detection of nodal metastases 5 mm or less in diameter. In addition, necrosis in nodes results in false-negative results and inflammation can cause false-positives [10,13]. In the first systematic review and meta-analysis on the use of whole-body ^18^F-FDG PET and ^18^F-FDG PET/CT in patients with vulvar cancer, Triumbari et al. reported that only 10 of 323 articles met all of the eligibility criteria [14]. The pooled data demonstrated a 76% sensitivity and 88% specificity for the detection of groin node metastases. This resulted in a 92% negative predictive value but only a 70% positive predictive value (PPV) in their patient population, which had a 28.6% prevalence of metastatic groin nodes. The review was limited by the relatively few original studies available and their heterogeneity regarding such things as histology and the stage of disease. They concluded that the PPV of PET/CT was limited and should be interpreted with caution. They suggested that larger prospective studies were required [14].

Ultrasound, combined with color Doppler, has the advantage of being able to look at the morphology of the nodes as well as the physical dimensions, but a single ultrasonic scan is not sufficient to guide management [10,15]. Ultrasound is often combined with fine-needle aspiration cytology for patients with suspicious findings, but Garganese et al. reported that the addition of fine-needle aspiration cytology did not increase the accuracy. They concluded that ultrasound alone was sufficient for lymph nodal staging in vulvar cancer [16]. In a retrospective study on 60 patients undergoing a preoperative groin ultrasound, de Gregoria et al. reported positive and negative predictive values of 82.9 and 87.5%, respectively [17]. A recent systematic review and meta-analysis, which included 437 patients and 914 groins, found that ultrasound had a high sensitivity (0.85; 95% CI: 0.81–0.89) and high negative predictive value (0.92; 95% CI: 0.91–0.94) [18].

Clinically palpable recurrence in an undissected groin carries a mortality of about 90% [19]. A long-term follow-up of the original GROINSS-V-1 study indicated that all seven patients who had a false-negative sentinel node died of disease after a routine clinical follow-up [20]. Recently, Pouwer et al. reported their results of adding ultrasonic surveillance of the groin for 76 patients who had a negative sentinel node biopsy. Their three-monthly follow-up visits consisted of groin palpation combined with an ultrasonic groin examination [21]. Two isolated asymptomatic groin recurrences were detected within 8 months, although one was clinically palpable. Both were treated with inguinofemoral lymphadenectomy and adjuvant radiation, and both were alive without evidence of disease at 39 and 120 months, respectively.

There is no survival benefit, but significant morbidity and use of resources, when lymph nodes that do not contain metastases are removed; however, detecting lymph node metastases early enough to still be able to cure the patient is critical. We hypothesized that this would be possible if serial groin ultrasounds were undertaken to detect nodal metastases before they became clinically palpable.

We undertook a pilot study in which we followed selected patients, including two patients who refused groin node dissection, through serial groin ultrasonography without any nodal dissection, for 12 months. Clinical follow-up was continued thereafter.

## 2. Materials and Methods

A prospective pilot study was performed at the Royal Hospital for Women in Sydney between September 2009 and October 2018, following approval from the South Eastern Sydney Local Health District Human Research Ethics Committee (reference number: 15/151(LNR/POWH/311)). Two patients from Brisbane were added to the study. One had a large squamous cell carcinoma and declined a groin node dissection, and the other had a small midline lesion, but a sentinel node was identified only on one side.

All patients initially had a baseline bilateral groin ultrasound. If this was negative, patients were offered standard surgical management of their primary cancer and 3-monthly ultrasonic surveillance of their groin nodes for 12 months, rather than sentinel node biopsy or groin node dissection, if their cancer met one of the following inclusion criteria: (1) A unifocal squamous cell carcinoma up to 20 mm in diameter, with > 1 mm stromal invasion. If the cancer was ipsilateral the patient had ipsilateral groin ultrasonography, but if it encroached within 1 cm of the midline bilateral ultrasonic surveillance was carried out. (2) An ipsilateral squamous cell carcinoma of any size which extended to within 1 cm either side of the midline. These patients were treated with ipsilateral inguinofemoral lymphadenectomy (IF LND) and ultrasonic surveillance of the contralateral groin. (3) Multifocal invasive lesions with the largest individual focus 20 mm or less in diameter. There were 3 additional patients: two patients refused groin dissection, including the one mentioned above from Brisbane, and a final patient was considered unfit for groin node dissection after the resection of the primary cancer.

Ultrasonic surveillance was considered to be successful if the scan revealed a metastasis in a lymph node (i.e., the patient had a positive scan) at the 3-monthly follow-up visit, even if a node was palpable, provided the previous ultrasonic scan was a true-negative upon review. All positive nodes upon ultrasound were removed and proven to contain metastatic disease histologically.

One patient was excluded from the study. She refused groin dissection and was followed by ultrasonic surveillance. She developed a positive node on ultrasound at 6 months, but again refused groin dissection or radiation therapy. She died 8 months later. No patients were lost to follow-up.

Following vulvar surgery, follow-up visits were at 3-monthly intervals for the first 12 months and consisted of a clinical examination together with a groin ultrasound. Some of these patients came from rural areas, and alternate follow-up visits, including clinical examinations and ultrasounds, were performed in their local towns. After 12 months, ultrasonic surveillance was not routinely performed, but clinical follow-up continued three-monthly until 2 years, 6-monthly until 5 years, and annually thereafter.

Most ultrasonic examinations were carried out at the Royal Hospital for Women. They were performed transcutaneously by using a Canon Aplio 600 system (Canon Medical Systems—Tochigi, Japan) with a 7–12 mHz linear transducer. Color and power Doppler assessments were routinely performed. The femoral triangle was assessed from its apex back towards the inguinal ligament. Deep and superficial nodes were assessed.

**Figure 1:**
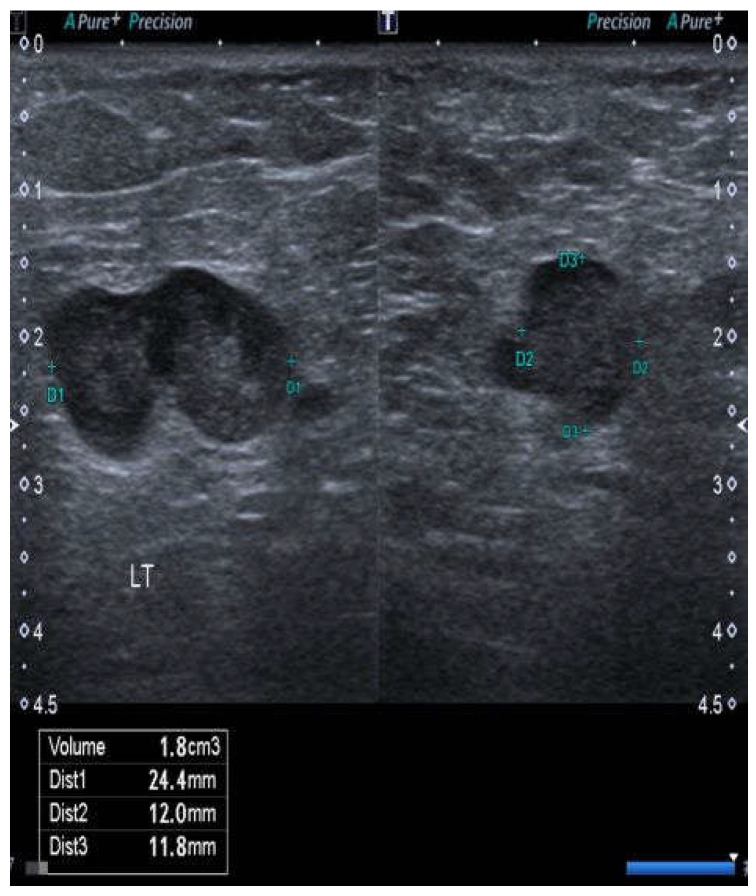
Case no. 10. Abnormal node from the right groin showing a lobulated surface contour of the cortex (or possibly two adherent nodes), interruption of the interface between the cortex and medulla, and a rounded rather than ovoid shape. All of these features suggest the tumor infiltration of the node.

Lymph nodes were considered suspicious if there was generalized or focal cortical thickening, any inhomogeneity of the texture of either the cortex or medulla, the absence of the medulla or hilum, focal masses within the cortex deforming or disrupting the junction with either the medulla or capsule, evidence of lymph node matting, or any evidence of an abnormal vascular pattern upon Doppler (Figure 1). If a node was more than 2.5 cm in length, particular attention was paid to any abnormal morphological features.

### Statistical Considerations

The primary outcome of the study was the accuracy of serial groin ultrasonography for the detection of positive nodes. Sensitivity of the surveillance via 3-monthly ultrasonography was calculated as the proportion of true-positive ultrasonic scans (confirmed histologically) of the total number of true-positive and false-negative scans. Similarly, the specificity was calculated as the proportion of true-negative scans of the total number of true-negative and false-positive scans. A false-positive scan was determined after fine-needle cytology was negative; two nodes were surgically removed and found to be negative histologically.

The positive predictive value (PPV) was the proportion of true-positive scans of the total number of true-positive and false-positive scans. The negative predictive value (NPV) was the proportion of true-negative scans of the total number of true-negative and false-negative scans. The 95% confidence intervals were calculated by using the method of Wilson [22].

Ultrasonic surveillance was considered to have failed if the scan was negative despite palpable positive nodes. The duration of follow-up was defined as the interval between the date of the vulvar and groin surgery and the date of the last follow-up.

The likelihood of finding a positive node at 24 months was calculated by using the method of Quigley [23].

## 3. Results

Overall, 32 patients were enrolled in the study. Their median age was 64.5 years (range of 35 to 88 years) and the median follow-up time was 37 months (range of 22 to 96 months)

Fourteen patients met criterion (1) (Table 1). This included one patient with a midline cancer in whom only one sentinel node could be identified. Their cancers had a median diameter of 10 mm (range of 6 to 20 mm), and a median depth of invasion of 1.9 mm (range of 1.2 to 3.5 mm).

Three of the fourteen patients (21.4%) had a positive node on serial ultrasonography. Of the three patients, two had clinically non-palpable groin nodes. These patients were diagnosed at 4 and 8 months, respectively. They were treated by unilateral inguinofemoral lymphadenectomy (IF LND) and bilateral groin as well as pelvic radiation; both remained alive and well at 96 and 36 months, respectively. One patient (7%) had a clinically palpable node at the time of ultrasonic diagnosis at 11 months, following prior negative ultrasonography. In spite of unilateral IF LND and bilateral groin as well as pelvic radiation, she died of disease 6 months later.

**Figure 2:**
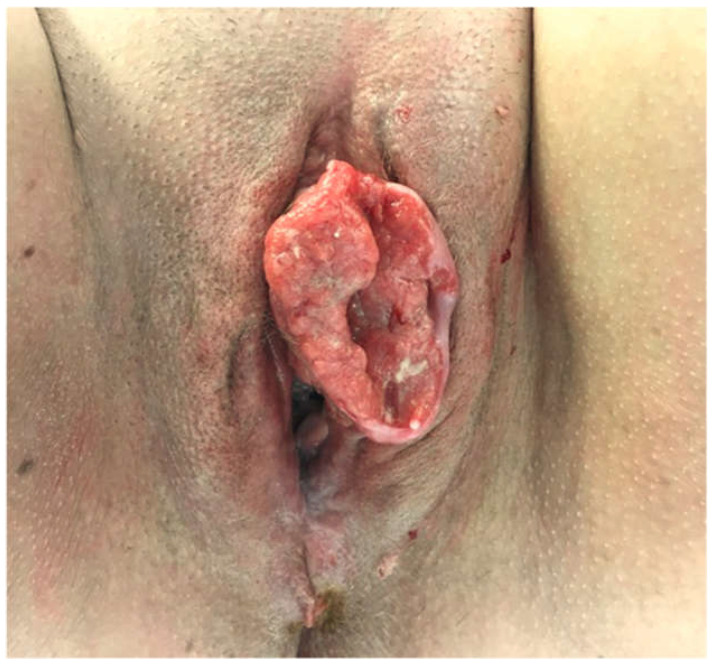
Case no. 20. A patient with a left unilateral 55 mm × 30 mm squamous cell carcinoma of the vulva, extending to the midline anteriorly. She was treated via radical local excision of the primary cancer, left inguinofemoral lymphadenectomy, and serial ultrasonography of the right groin.

There were 10 patients who met criterion (2) (Figure 2) (Table 2). These patients were treated with ipsilateral IF LND and ultrasonic surveillance of the contralateral groin. Their cancers had a median largest diameter of 45.5 mm (range of 18 mm to 60 mm) and a median depth of invasion of 7.6 mm (range of 3.4 to 19 mm). Two of these patients (20%) had cancers that would have been suitable for bilateral sentinel node biopsy, but both elected to have unilateral IF LND and contralateral ultrasonic surveillance. Three of the patients (30%) had positive ipsilateral inguinal nodes but none developed a contralateral metastasis, with follow-up from 30 to 74 months. 

The two patients with one positive ipsilateral node received no adjuvant therapy, but the patient with two positive nodes received bilateral groin as well as pelvic radiation. All three remained alive and free of disease from 30 to 48 months.

Five patients met criterion (3), having small multifocal invasive lesions following the resection of apparent in situ disease. Three of these patients had Paget’s disease, and two had squamous cell carcinomas (Table 3).

Three other patients were followed with serial ultrasonography (Table 4). Two patients with squamous cell carcinomas refused any form of groin node dissection. The third patient was considered unfit for groin surgery.

The overall sensitivity of ultrasonic surveillance was 100% (95% CI: 44–100%), with a specificity of 97% (95% CI: 83–99%). The positive predictive value was 75% (95% CI: 31–95%) and the negative predictive value was 100% (95% CI: 88–100%).

## 4. Discussion

To our knowledge, this is the first report of the use of serial groin ultrasonography in lieu of sentinel node biopsy or inguinofemoral lymphadenectomy for the management of selected patients with vulvar cancer. Ultrasonic surveillance was used to follow 43 groins in 32 patients. Positive nodes were identified upon ultrasound in three patients (9.4%) and one patient died of disease (3.1%). This patient had a clinically palpable node at the time of ultrasonic diagnosis, and a review of the previous ultrasound confirmed it to be negative. Thirty-nine groins (90.7%) were preserved intact.

In this pilot study, there was 100% sensitivity and 100% negative predictive value for 32 patients, with a median follow-up of 37 months, (range of 22 to 96 months). One patient had a false-positive scan, giving a specificity of 97% (95% CI: 88–100%). The encouraging outcome of this pilot study justifies a larger, multicenter study to see if these results can be replicated.

Motivation for this study came in 2009, when we saw a patient with a 6 × 6 mm squamous cell carcinoma of the vulva with 1.2 mm of stromal invasion. The risk of positive nodes was considered to be so small that even sentinel node biopsy seemed unnecessary. Instead, we discussed with the patient the possibility of following her with serial groin ultrasonography for 12 months. She was happy with this decision, and as everything went smoothly it was decided to seek ethics approval for a pilot study on three groups of patients.

The first group of 14 patients all had cancers which would have been suitable for sentinel node biopsy. The latter is recommended for lesions 4 cm or less in diameter with more than 1 mm of stromal invasion, but we restricted our criteria to lesions up to 2 cm in diameter and more than 1 mm invasion in this pilot study. Three patients (21.4%) developed a positive node during follow-up. Two of these patients had non-palpable nodes. As we had hypothesized, both were cured of their disease after unilateral inguinofemoral lymphadenectomy and bilateral groin as well as pelvic radiation. The third patient had a midline lesion. At the time of her positive ultrasonic scan, which was 11 months post-primary surgery, she had asymptomatic, palpable nodes bilaterally. At bilateral inguinofemoral lymphadenectomy she had three positive nodes on one side and one positive node on the other side. The largest node was 29 mm diameter, and all four positive nodes had extracapsular spread. In spite of bilateral groin and pelvic radiation, she died 6 months after groin dissection.

The second group had a unilateral cancer that extended to within 1 cm either side of the midline. There were 10 patients in this group; the median diameter of their cancers was 45.5 mm. Three of these patients (30%) had positive ipsilateral nodes, but none developed a positive contralateral node, so all 10 had the complete preservation of their contralateral nodes.

The third group was patients who were thought to have mainly VIN or in situ Paget’s disease but were found to have multifocal small, invasive foci when the primary disease was excised. The largest individual focus of invasive cancer was 16 mm in diameter. No patient developed evidence of nodal metastases, and eight groins were preserved intact.

There were three additional patients, two of whom refused groin dissection. One of these patients had a false-positive scan, and so had two nodes removed from one side. The third patient was 87 years of age and had advanced systemic lupus erythematosus. She was considered unfit for groin surgery after undergoing modified radical vulvectomy for the primary lesion.

With the recent use of serial ultrasonography to monitor patients with a negative sentinel node [21], sentinel node biopsy should be universally regarded as the treatment of choice for patients with a unifocal vulvar cancer of up to 4 cm or less in diameter [24]. Sentinel nodes were initially identified with a blue dye and radiocolloid lymphoscintigraphy, but since 2010 there has been increasing use of infrared imaging with indocyanine green, alone or in combination with other methods. Even when all methods were combined, failure to detect sentinel nodes was reported in 4% of cases at Memorial Sloan-Kettering Cancer Center [25].

Ultrasound alone is considered to be the imaging method of choice for the preoperative assessment of inguinal lymph nodes [16,18,26]. An advantage of ultrasound is that it is cost-effective, easy to learn, easy to perform, and does not require the use of radiation or contrast agents [16]. This means that its use does not need to be limited to tertiary treatment centers. In fact, the patient with the first positive node that we identified in this study had her ultrasound performed in a rural town. The node was not clinically palpable at the time. After unilateral inguinofemoral lymphadenectomy and bilateral groin as well as pelvic radiation, the patient remains alive without groin recurrence at 96 months.

The critical issue is the size that a metastasis must be before it can be detected via ultrasound. In this pilot study, the smallest metastatic deposit that was detected was 5 mm.

The rarity of vulvar cancer and the lack of standardized ultrasonic nomenclature to describe the morphological features of lymph nodes have made it difficult to establish reliable criteria with which to identify suspicious lymph nodes [18]. Garganese et al. reported that the combination of two ultrasonic parameters (the short-axis (S) length and cortex/medulla thickness ratio of the dominant lymph node) produced the greatest accuracy for discriminating between negative and positive lymph nodes (sensitivity of 88.9%, specificity of 82.4%) (16). In our study, cortical abnormalities such as thickening, a focal abnormality, or the disruption of the junction between the cortex and medulla were the most common abnormalities.

The consensus opinion from the Vulvar International Tumor Analysis (VITA) group was recently published [26]. This group was created in 2016 with the aim of providing a consensus opinion on the appropriate examination technique, measurement technique, and terminology to be used to describe the ultrasonic appearance of inguinal lymph nodes in future studies on women with vulvar cancer. The agreement between these ultrasonic features and the histologic diagnosis has yet to be established.

Two important issues in relation to serial groin ultrasonography are the frequency with which it should be performed and the duration for which it should be continued.

In both this pilot study, using serial ultrasonography in lieu of any form of groin dissection, and in the Dutch study, which followed patients with a negative sentinel node(s), 3-monthly ultrasounds were performed. Between the two studies, five patients with positive nodes were identified. Three of these nodes were not clinically palpable, and all three patients (100%) were cured after groin node dissection and bilateral groin as well as pelvic radiation; however, two patients had clinically palpable nodes at the time of the positive ultrasonic diagnosis, and one of these patients (50%) died of disease. This is consistent with data from the literature, which indicate that about 90% of patients who recur in an undissected groin will die of disease when patients are followed clinically [19]. These data strongly suggest that ultrasonic surveillance should be performed at least every 2 months.

In an earlier study on 60 patients with vulvar cancer from our institution, Farrell et al. investigated patient preferences with respect to lymphedema or sentinel node biopsy followed by clinical follow-up [27]. Patients were told that inguinofemoral lymphadenectomy would be associated with a 50% risk of chronic lower limb lymphedema, while a sentinel node biopsy would be associated with a 5% false-negative rate and that these patients would have a 90% mortality rate. Although 73% of the patients actually had lymphedema, 53% (32 of 60) said they would be prepared to take no risk of death at all, and a further 10% said they would take a 1 in 1,000,000 risk. These data indicate that the majority of patients are not prepared to take any risk with their lives, and we believe they would readily accept a 2-monthly scan if the advantages and disadvantages were explained to them.

With respect to the appropriate duration of ultrasonic surveillance, we discontinued it after 12 months, whereas the Dutch study continued it until 24 months [21]. If the patient we excluded for refusing treatment of her positive node is considered, there were six positive nodes diagnosed by ultrasound. Two were diagnosed at 4 months, one at 6 months, two at 8 months, and one at 11 months. Although no positive nodes were discovered after 12 months, we have determined that, statistically, the true likelihood of discovering a positive node at 24 months would not exceed 1.6%, based on the number of patients with 24 months of follow-up available in this pilot study [23].

Strengths of this study are that all data were collected prospectively, no patient was lost to follow-up, and no restrictions were placed on the venue for the ultrasonic surveillance. The main limitation is the relatively small number of patients.

In summary, the high sensitivity and negative predictive value reported in this pilot study suggest that further evaluation of the concept of serial groin ultrasonography in lieu of some form of groin dissection would be warranted in selected patients with vulvar cancer. An observational study with stopping rules could be undertaken. Encouraged by the results of this pilot study, we would suggest that, for patients in the first category, the diameter of the cancer could be increased from 2 to 4 cm, making it consistent with criteria for sentinel node biopsy [24].

We believe that the interval between scans should be no longer than 2 months, given the fact that two of the six nodes detected by ultrasonic surveillance in this study and that of Pouwer et al. [21] were clinically palpable at the time of diagnosis. It is hoped that, with more frequent scanning, most positive lymph nodes would be detected before they became clinically palpable, when the cure rate is high. 

## 5. Conclusions

Serial groin ultrasonography in lieu of sentinel node biopsy or inguinofemoral lymphadenectomy appears to be a reasonable approach for selected patients with vulvar cancer. If these findings can be replicated in a larger prospective study, this approach would allow many patients to be cured of their disease without the need for any type of groin dissection and its associated morbidity, particularly chronic lower limb lymphedema.

## Figures and Tables

**Table 1:** Patients with a squamous cell carcinoma of the vulva that was 20 mm or less in diameter with >1 mm stromal invasion, who had ipsilateral or bilateral ultrasonic surveillance rather than sentinel node biopsy.

Patient Number	Dimensions of the Primary Cancer	Nodes	Outcome Months
1	Unilateral 6 × 6 mm. Invasion 1.2 mm.	Neg	NED 88
2	Midline 8 × 6 mm. Invasion 2.2 mm.	Neg	NED 65
3	Unilateral 9 × 9 mm. Invasion 2.7 mm.	Pos	NED 96
4	Unilateral 15 ×15 mm. Invasion 1.7 mm.	Neg	NED 49
5	Unilateral 10 × 10 mm. Invasion 1.7 mm.	Neg	NED 36
6	Midline 8 × 8 mm. Invasion 1.3 mm.	Neg	NED 34
7	Midline 15 × 15 mm. Invasion 1.4 mm.	Neg	NED 26
8	Midline 12 × 7 mm. Invasion 2.5 mm.	Neg	NED 24
9	Unilateral 8 × 6 mm. Invasion 2.2 mm.	Neg	NED 23
10	Midline 15 × 15 mm. Invasion 3.5 mm.	Pos	DOD 6 *
11	Unilateral 10 × 10 mm. Invasion 2 mm.	Neg	NED 22
12	Unilateral 6 × 6 mm. Invasion 2.4 mm.	Neg	NED 20
13	Midline 20 ×12 mm. Invasion 3.0 mm.	Neg	NED 18
14	Midline 20 × 20 mm. Invasion 1.8 mm. Unable to identify one sentinel node.	Pos	NED 36

* Time to death from the treatment of positive nodes. NED: no evidence of disease.

**Table 2:** Patients with a unilateral squamous cell carcinoma of the vulva extending to within 1 cm of, or just beyond, the midline, who had ipsilateral inguinofemoral lymphadenectomy and ultrasonic surveillance of the contralateral groin.

Patient Number	Status of Primary Cancer	Ipsilateral Nodes	Outcome Months
15	18 × 18 mm. Invasion 4.3 mm.	11 neg	NED 65
16	46 × 20 mm. Invasion 3.4 mm.	14 neg	NED 74
17	45 × 30 mm. Invasion 5.8 mm.	12 neg	NED 69
18	18 × 18 mm. Invasion 4.3 mm.	11 neg	NED 72
19	50 × 40 mm. Invasion 7.2 mm.	1/12 pos	NED 48
20	55 × 30 mm. Invasion 8 mm.	4 neg	NED 41
21	60 × 60 mm. Invasion 14 mm.	2/10 pos	NED 38
22	45 × 55 mm. Invasion 9 mm.	12 neg	NED 36
23	45 × 30 mm. Invasion 19 mm.	13 neg	NED 32
24	50 × 30 mm. Invasion 8 mm.	1/7 pos	NED 30

NED: no evidence of disease.

**Table 3:** Patients with mainly in situ carcinomas that demonstrated small, multifocal invasive disease on resection. The largest individual invasive focus was 20 mm or less in diameter.

Patient Number	Status of Primary Cancer	Nodes	Outcome Months
25	Bilateral in situ and invasive Paget’s disease. Largest invasive focus 8 × 3 mm. Invasion 2.8 mm.	Neg	NED 72
26	Bilateral in situ and invasive SCCs. Largest invasive focus 5 mm. Invasion 1.9 mm.	Neg	NED 49
27	Bilateral in situ and invasive SCCs. Largest invasive focus 14 × 12 mm. Invasion 3.3 mm.	Neg	NED 42
28	Unilateral in situ and invasive Paget’s disease. Largest invasive focus 16 × 16 mm. Invasion 3 mm.	Neg	NED 39
29	Unilateral in situ and invasive Paget’s. Largest invasive focus 12 × 6 mm. Invasion 2 mm.	Neg	NED 32

NED: no evidence of disease; SCC: squamous cell carcinoma.

**Table 4:** Patients with squamous carcinomas of the vulva who either refused or were considered unfit for groin node dissection and were followed by ultrasonic surveillance of the groin(s).

Patient Number	Status of Primary Cancer	Nodes	Outcome Months
30	Ipsilateral SCC, 50 × 30 mm. Invasion 3.5 mm (refused).	Neg	NED 60
31	Multifocal SCC. Largest focus 30 × 20 mm. Invasion 1.3 mm (refused).	False pos at 10 months	NED 26
32	Ipsilateral SCC 56 × 35 mm. Invasion 19 mm (unfit).	Neg	NED 16

SCC: squamous cell carcinoma; NED: no evidence of disease.

## Data Availability

The data used to support the findings of this study have not been made available because they are restricted by the Ethics Committee in order to protect patient confidentiality.
